# The Biological Cost of Every Heartbeat: Imaging-Derived Cardiovascular Vulnerability in Infective Endocarditis

**DOI:** 10.3390/ijms27062733

**Published:** 2026-03-17

**Authors:** Corina-Ioana Anton, Rareș Constantin Ranetti, Adrian Streinu-Cercel

**Affiliations:** 1 Department of Infectious Diseases, “Dr. Carol Davila” Central Military Emergency University Hospital, Calea Plevnei 134, 010242 Bucharest, Romania; corina-ioana.anton@drd.umfcd.ro; 2 Department of Medico-Surgical and Prophylactic Disciplines, Titu Maiorescu University, 040441 Bucharest, Romania; 3 Faculty of General Medicine, Carol Davila University of Medicine and Pharmacy, Bulevardul Eroii Sanitari 8, 050474 Bucharest, Romania; adrian.streinucercel@umfcd.ro; 4 Department of Infectious Diseases I, Faculty of Medicine, Carol Davila University of Medicine and Pharmacy, 020021 Bucharest, Romania; 5 National Institute for Infectious Diseases “Prof. Dr. Matei Balş”, Strada Doctor Calistrat Grozovici 1, 021105 Bucharest, Romania

**Keywords:** biological age, coronary flow reserve, echocardiography, infective endocarditis, screening

## Abstract

Biological cardiovascular vulnerability is defined as an imaging-derived construct integrating myocardial functional impairment, coronary microvascular dysfunction, and modeled hemodynamic burden, including global longitudinal strain, coronary flow reserve, and derived vascular indices. To evaluate whether advanced echocardiographic and coronary Doppler imaging parameters identify biological cardiovascular vulnerability associated with the severity and complications of infective endocarditis beyond conventional structural findings. In this retrospective single-center cohort study, we analyzed consecutive patients with definite infective endocarditis who underwent advanced echocardiographic and coronary Doppler imaging. Comprehensive transthoracic and transesophageal echocardiography assessed vegetation characteristics, left ventricular function, global longitudinal strain (GLS), diastolic indices, right ventricular function, and pulmonary artery systolic pressure. Coronary microvascular function was evaluated noninvasively using transthoracic Doppler-derived coronary flow reserve (CFR) of the left anterior descending artery. Associations with disease severity and perivalvular complications were evaluated using multivariable regression analysis. Reduced coronary flow reserve was independently associated with the composite severe infective endocarditis phenotype, as defined by perivalvular complications, severe valvular dysfunction, or endocarditis team-guided urgent surgical indication. Coronary flow reserve correlated inversely with vegetation size (r = −0.39; *p* = 0.002) and regurgitation severity (r = −0.36; *p* = 0.004). Notably, the inverse association between coronary flow reserve and vegetation size showed substantial interindividual variability, particularly among patients with similar vegetation dimensions, suggesting heterogeneity in microvascular vulnerability beyond structural lesion burden. Despite relatively preserved mean arterial pressure across age groups, advanced imaging revealed progressive increases in systemic vascular resistance, declining wall shear stress, impaired microvascular flow, and reduced myocardial reserve. Imaging-derived cardiovascular vulnerability profiles frequently diverged from chronological age, highlighting heterogeneity in cardiovascular reserve despite apparently stable conventional hemodynamic parameters. Advanced echocardiographic and coronary Doppler imaging characterize a spectrum of biological cardiovascular vulnerability that is associated with clinically adjudicated severity in infective endocarditis, rather than serving as independent prognostic predictors.

## 1. Introduction

Infective endocarditis (IE) remains a life-threatening cardiovascular disease with substantial morbidity and mortality, despite advances in antimicrobial therapy and multimodal imaging [1]. Contemporary diagnostic strategies primarily rely on clinical suspicion, microbiological confirmation, and echocardiographic detection of structural abnormalities, particularly valvular vegetations [2]. Although effective for confirming established disease, this approach frequently identifies IE only after significant valvular destruction, embolic events, or perivalvular extension have occurred [2].

Echocardiography plays a central role in the diagnosis and management of IE, with transthoracic echocardiography as the first-line modality and transesophageal echocardiography providing superior detection of vegetations and complications [3]. However, conventional echocardiographic assessments remain largely focused on anatomical findings. Structural imaging alone does not fully explain the marked heterogeneity in disease severity, progression, and outcomes observed in patients with IE [4]. Recent studies in heart failure and systemic inflammatory states have highlighted the importance of functional reserve and microvascular integrity as determinants of clinical outcomes, supporting the concept that cardiovascular vulnerability may precede overt structural deterioration [4].

In infective endocarditis, functional and microvascular imaging parameters may also be influenced by systemic factors such as sepsis-related myocardial depression, anemia, tachycardia, and inflammatory cytokine activation, which can transiently impair myocardial performance and coronary microvascular function.

Notably, an increasing proportion of patients develop severe IE in the absence of classical predisposing conditions, such as congenital heart disease, prosthetic valves, or prior endocarditis [2,3]. This suggests that subclinical cardiovascular vulnerability may precede overt structural damage. Functional myocardial impairment, altered hemodynamic loading, endothelial dysfunction, and microvascular abnormalities may influence the biological environment in which the infection develops and progresses [3].

Advanced echocardiographic techniques provide the opportunity to move beyond purely structural assessments [2,3]. Global longitudinal strain (GLS) enables the sensitive detection of subclinical myocardial dysfunction, whereas Doppler-derived indices of diastolic and right ventricular function offer insights into ventricular reserve and pulmonary vascular load [4]. In parallel, transthoracic Doppler assessment of coronary flow reserve allows for the noninvasive evaluation of coronary microvascular function, reflecting the integrated effects of endothelial health, inflammatory burden, and blood rheology [5]. These imaging-derived parameters may identify biological cardiovascular vulnerabilities that are not apparent from conventional vital signs or standard echocardiographic metrics [4,5].

Importantly, conventional hemodynamic measures, such as blood pressure, may remain relatively preserved even as vascular resistance increases, shear stress declines, and microvascular flow deteriorates with advancing biological cardiovascular aging [6]. This dissociation may obscure clinically relevant vulnerabilities, delaying the recognition of patients at risk for severe IE and its complications [7]. Imaging-based functional and microvascular markers may therefore provide a more sensitive assessment of the cumulative cardiovascular burden than chronological age or structural findings alone and may inform the earlier use of transesophageal echocardiography and intensified surveillance strategies [8].

We hypothesized that the severity and complications of infective endocarditis are associated with imaging-derived biological cardiovascular vulnerability, defined by the integration of structural, functional, hemodynamic, and microvascular echocardiographic parameters.

This study aimed to characterize whether advanced echocardiographic and coronary Doppler imaging parameters are associated with a severe infective endocarditis phenotype, as defined by structural complications and multidisciplinary clinical decision-making, beyond conventional structural assessment.

## 2. Results

A total of 143 patients with definite infective endocarditis were included in the final analysis. Baseline demographic and clinical characteristics are summarized in Table 1.

Age group cutoffs were selected to reflect clinically meaningful life stages and to ensure balanced subgroup sizes across the wide age distribution of the cohort for exploratory comparison.

**Figure 1:**
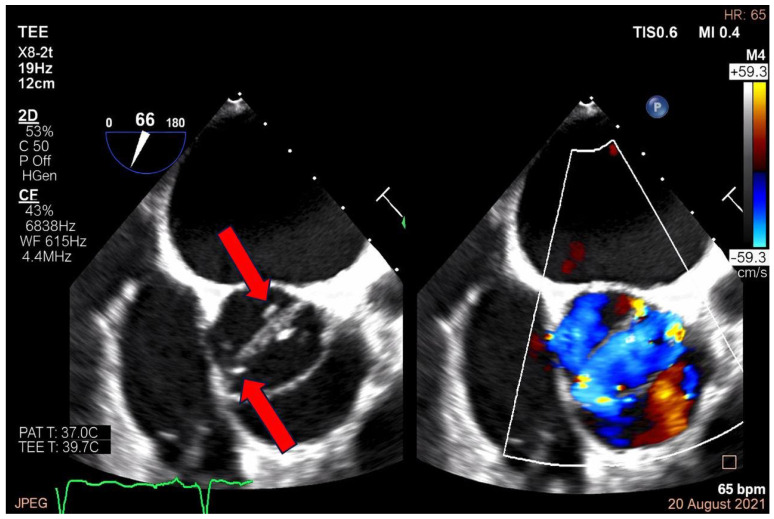
Transesophageal echocardiography images demonstrating valvular involvement in infective en-do-carditis. **Left panel**: two-dimensional TEE view showing irregular echogenic masses attached to the valve leaflets (red arrows), consistent with valvular vegetations. **Right panel**: color Doppler imaging demonstrating turbulent regurgitant flow across the affected valve. In the color Doppler map, red indicates blood flow toward the transducer and blue indicates flow away from the transducer, while the mosaic pattern (red–blue–yellow) reflects turbulent flow, suggestive of significant valvular regurgitation associated with the infectious process.

The study flow is shown in Figure 1. A proportion of patients presented with clinical signs of heart failure at admission, which may have influenced baseline functional and microvascular imaging parameters.

The median age of the study population was 67 years (interquartile range 59–74 years; range 23–95 years), reflecting a heterogeneous cohort spanning young adulthood to advanced age.

Patients with a severe infective endocarditis phenotype had a higher prevalence of several baseline comorbidities compared with those with non-severe disease, including diabetes mellitus, chronic kidney disease, prior heart failure, atrial fibrillation, and prior valvular heart disease (Table 2). Coronary artery disease and chronic obstructive pulmonary diseases were also more frequent in the non-severe group. These findings highlight differences in baseline clinical profiles.

Baseline demographic, clinical, and laboratory characteristics of the study population are summarized in Table 3. The cohort had a median age of 67 years (interquartile range 59–74) and a median body mass index of 26.1 kg/m^2^ (IQR 23.4–29.8). Established risk factors for infective endocarditis were common, including intravenous drug use in 20% of patients and the presence of a prosthetic valve in 36%. HIV infection and chronic immunosuppression were infrequent.

Cardiovascular and non-cardiovascular comorbidities were prevalent, with hypertension, diabetes mellitus, coronary artery disease, atrial fibrillation, and chronic kidney disease frequently observed. Laboratory evaluation at admission demonstrated elevated inflammatory markers, anemia, and leukocytosis, consistent with active systemic infection. Metabolic and endocrine parameters showed heterogeneous profiles across the cohort.

Structural echocardiographic findings are detailed in Table 4. The mean maximal vegetation diameter was 1.58 ± 0.67 cm (range 0.31–2.47 cm). Single vegetations were identified in 61% of patients, two vegetations in 29%, and three or more vegetations in 10%. Vegetation mobility was classified as highly mobile in 52%, moderately mobile in 35%, and fixed in 13% of cases. Echogenicity analysis demonstrated hyperechogenic vegetations in 45%, mixed echogenicity in 38%, and hypoechogenic vegetations in 17%.

Representative transesophageal echocardiographic imaging of aortic valve vegetation is shown in Figure 1.

The global cardiac function and hemodynamic parameters are summarized in Table 5. The mean left ventricular ejection fraction was 48 ± 13%, while the global longitudinal strain (GLS) demonstrated impaired myocardial deformation (−16.9 ± 3.5%). Diastolic indices showed elevated filling pressures, with a mean E/e′ ratio of 15.8 ± 7.1 and indexed left atrial volume of 42 ± 15 mL/m^2^. Right ventricular systolic function was reduced, with a mean TAPSE of 18.7 ± 4.7 mm, and the pulmonary artery systolic pressure was elevated (44 ± 17 mmHg).

**Figure 2:**
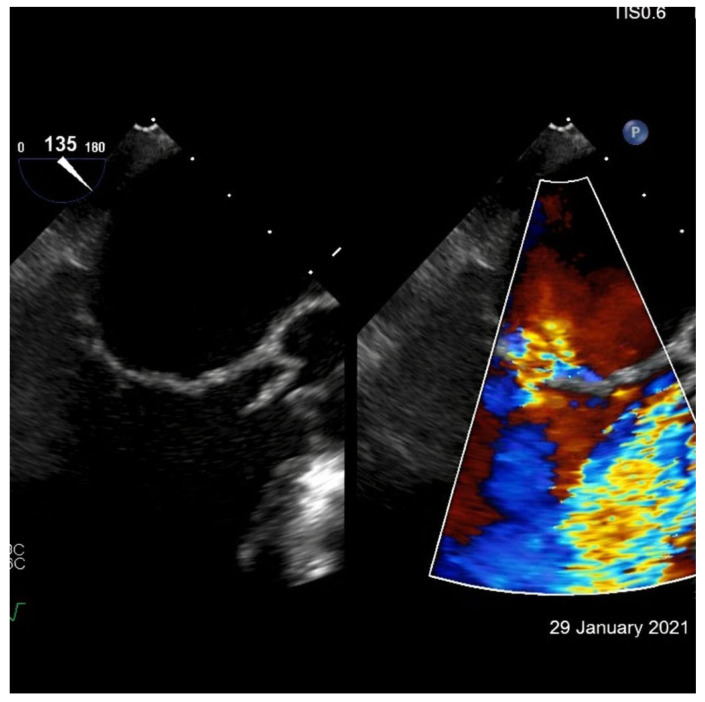
Transesophageal echocardiographic assessment of cardiac hemodynamics. Representative mid-esophageal transesophageal echocardiographic long-axis view with corresponding color Doppler imaging. The grayscale image depicts global ventricular morphology, while color Doppler demonstrates marked intracardiac flow turbulence during diastole, reflecting altered ventricular filling dynamics. These qualitative findings are concordant with impaired left ventricular systolic function, abnormal myocardial deformation, and elevated filling pressures as quantified by left ventricular ejection fraction, global longitudinal strain, and diastolic indices.

GLS correlated inversely with E/e′ (r = −0.58; *p* < 0.001). Representative transesophageal echocardiographic images illustrating these findings are shown in Figure 2.

Coronary Doppler-derived microvascular parameters are shown in Table 6. Mean left anterior descending artery flow was 44 ± 18 mL/s, and mean coronary flow reserve (CFR) was 2.2 ± 0.8. A CFR < 2.0, a commonly used reference threshold in prior literature, was observed in 32.1% of patients and was used to categorize relative impairment of coronary microvascular function within this cohort. CFR correlated inversely with vegetation size (r = −0.39; *p* = 0.002) and severity of valvular regurgitation (r = −0.36; *p* = 0.004).

**Figure 3:**
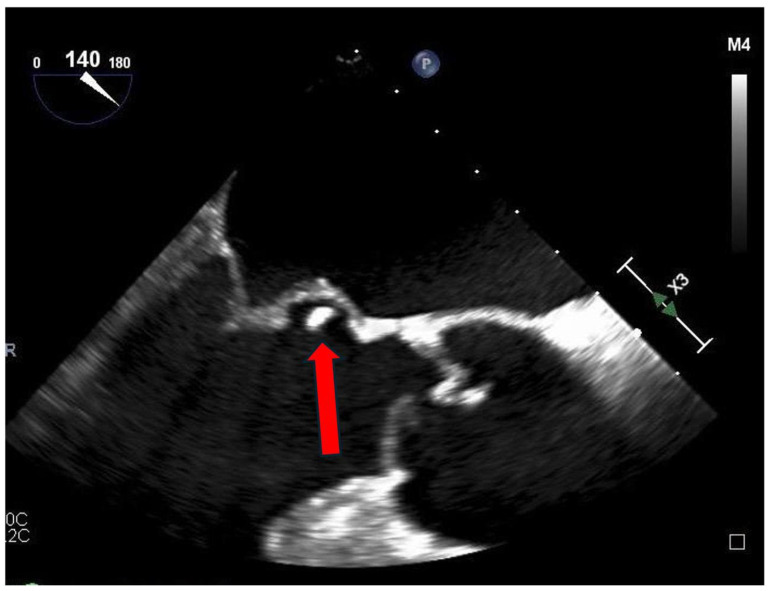
Transesophageal echocardiographic visualization of valvular vegetation. Mid-esophageal transesophageal echocardiographic long-axis view demonstrating an echogenic, irregular mass attached to the valvular apparatus, protruding into the cardiac lumen. The lesion is clearly distinguishable from adjacent valve tissue and is visualized along the line of leaflet coaptation, consistent with a mobile valvular aneurysm. The arrow indicates a mobile echogenic mass attached to the valve leaflet, consistent with valvular vegetation, a characteristic finding in infective endocarditis.

The perivalvular complications and their association with vegetation size are summarized in Table 7. Abscess formation occurred in 38% of patients, aneurysms in 25%, prosthetic dehiscence in 18%, and leaflet perforation in 14%. All complications were associated with larger vegetation dimensions, with mean sizes approaching or exceeding 2.0 cm. Representative transesophageal echocardiographic images of the valvular aneurysm are shown in Figure 3.

**Figure 4:**
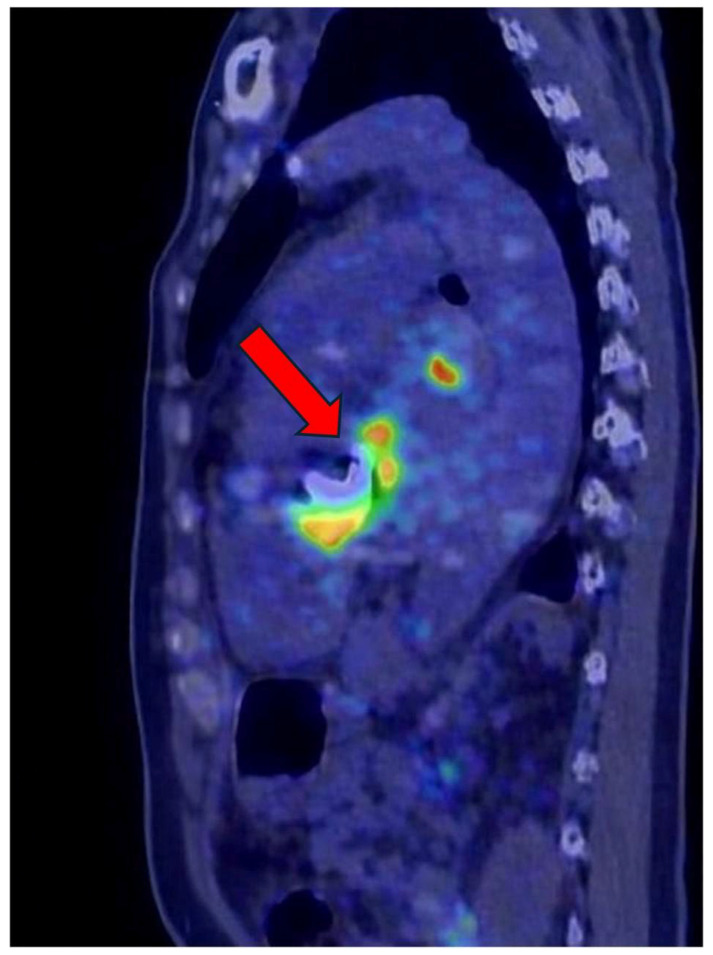
Sagittal ^18^F-FDG PET/CT fusion image showing focal increased metabolic activity at the level of the cardiac valve/perivalvular region (arrow). The intense radiotracer uptake (yellow–red signal) reflects inflammatory activity associated with infective endocarditis, supporting the presence of active infection.

**Figure 5:**
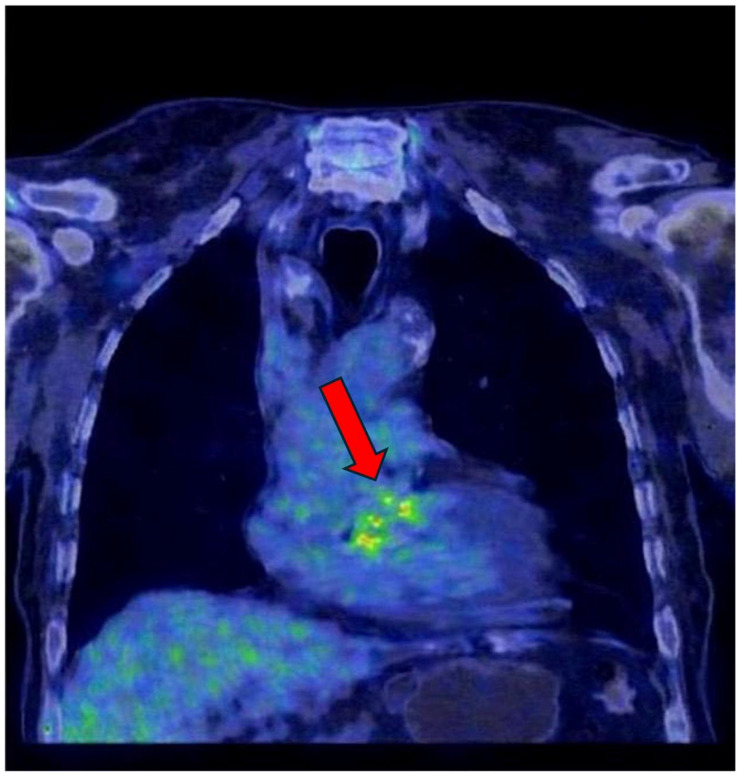
^18^F-FDG PET/CT fusion image demonstrating focal increased metabolic activity in the cardiac region (red arrow), consistent with inflammatory or infectious involvement associated with infective endocarditis.

In selected cases with suspected prosthetic involvement, ^18^F-fluorodeoxyglucose positron emission tomography/computed tomography (FDG PET/CT) was used as a complementary imaging modality. Sagittal and coronal PET/CT images demonstrated focal and heterogeneous periprosthetic FDG uptake, consistent with active inflammatory or infectious processes extending beyond the prosthetic annulus (Figure 4 and Figure 5). These metabolic abnormalities support the diagnosis of prosthetic valve dehiscence in the appropriate clinical and echocardiographic context and provide incremental information beyond structural echocardiographic findings, particularly in patients with complex perivalvular involvement.

Key correlations between imaging-derived parameters are presented in Table 8. Vegetation size correlated strongly with regurgitation severity and pulmonary artery systolic pressure, while CFR correlated inversely with both vegetation size and regurgitation severity.

Age-stratified analysis of systemic blood pressure demonstrated a relatively stable mean arterial pressure across age groups despite increasing systolic blood pressure with advancing age (Table 9). The mean arterial pressure values were clustered within a narrow range across decades, including in older patients, indicating the preservation of conventional perfusion pressure metrics at presentation. This stability occurred despite the progressive changes observed in the imaging-informed hemodynamic and microvascular parameters described below.

Systemic vascular resistance increased progressively with age (Table 10), despite relatively stable mean arterial pressure. This pattern is consistent with the modeling assumptions incorporating age-adjusted cardiac output estimates and illustrates a progressively higher calculated vascular load required to maintain similar perfusion pressure across age groups.

These findings highlight an age-related increase in vascular load that was not apparent from blood pressure measurements.

Calculated serum osmolarity increased progressively from young adulthood to the seventh decade of life, exceeding 300 mOsm/kg in patients aged 61–70 years (Table 11). In the older age groups, the osmolarity values plateaued or declined modestly. This nonlinear age-related pattern suggests heterogeneity in metabolic and fluid balance across decades within the infective endocarditis population.

Estimated wall shear stress declined steadily with advancing age (Table 12). Younger patients exhibited higher calculated shear stress values, while older age groups demonstrated progressively lower estimates. Estimated wall shear stress declined steadily with advancing age within the applied modeling framework, indicating lower calculated shear forces under assumptions of age-related changes in vascular geometry and flow.

Analysis of contrast transport dynamics revealed a progressive reduction in contrast velocity and prolongation of transit time with increasing age (Table 13). The derived contrast permeability index declined steadily across age groups, indicating reduced intravascular transport efficiency. These changes were observed consistently with advancing age and paralleled the deterioration seen in other imaging-informed hemodynamic markers.

Right ventricular and coronary Doppler parameters demonstrated progressive age-related deterioration (Table 14). Pulmonary artery systolic pressure increased steadily with age, while tricuspid annular plane systolic excursion declined, indicating reduced right ventricular systolic function. In parallel, left anterior descending coronary artery flow and coronary flow reserve decreased progressively, with CFR values falling below 2.0 in the oldest age groups, consistent with increasing impairment of coronary microvascular function.

All statistical findings should be interpreted in the context of exploratory analyses without adjustment for multiple testing.

## 3. Discussion

The definition of severe infective endocarditis used in this study incorporates both imaging findings and multidisciplinary treatment decisions, introducing incorporation bias. Consequently, the present analyses do not establish prognostic prediction but rather describe associations between advanced imaging parameters and a clinically relevant severity phenotype encountered in routine practice. This limits causal inference and precludes conclusions regarding the ability of these imaging markers to prospectively predict outcomes.

From a biological perspective, infective endocarditis is characterized by sustained systemic inflammation, endothelial activation, altered nitric oxide signaling, oxidative stress, and microcirculatory dysfunction. These molecular and cellular processes impair vascular reactivity, disrupt myocardial energetics, and reduce microvascular reserve.

Advanced echocardiographic and coronary Doppler parameters should therefore be interpreted as integrative imaging surrogates of these underlying biological pathways rather than as direct mechanistic measurements.

The baseline clinical and laboratory profile of the cohort highlights the substantial heterogeneity of host cardiovascular and systemic vulnerability in infective endocarditis.

Traditional risk factors, including intravenous drug use and prosthetic valve presence, coexisted with a high burden of cardiometabolic and renal comorbidities, as well as pronounced inflammatory activation at presentation. These baseline characteristics likely contribute to interindividual differences in cardiovascular reserve and may modulate functional and microvascular imaging parameters independently of valvular lesion burden.

By integrating advanced echocardiographic markers of myocardial deformation, coronary microvascular function, and imaging-informed hemodynamic indices, our findings highlight the cumulative biological cost of each heartbeat as a determinant of the clinical expression of infective endocarditis.

### 3.1. Principal Imaging Findings

The principal findings of this study are as follows: First, conventional structural markers, such as the size and mobility of vegetation, remain central determinants of disease severity and complications. Second, global longitudinal strain revealed substantial subclinical myocardial dysfunction despite only moderate reductions in left ventricular ejection fraction. Third, coronary microvascular dysfunction, as indicated by reduced coronary flow reserve, was commonly associated with both disease severity and perivalvular extension. Fourth, age-stratified imaging analyses revealed a consistent dissociation between preserved mean arterial pressure and the progressive deterioration of vascular resistance, wall shear stress, myocardial reserve, and microvascular transport efficiency.

Taken together, these findings suggest that infective endocarditis develops and progresses within a cardiovascular system that may already be biologically compromised, even when conventional clinical parameters appear stable.

Accordingly, imaging-derived measures of cardiovascular vulnerability should be interpreted in the context of underlying comorbidities and systemic inflammatory status rather than as isolated disease-specific markers.

### 3.2. Beyond Structural Imaging: Functional and Microvascular Vulnerability

Traditionally, echocardiography in IE has focused on the anatomical detection of vegetations and complications [8]. Although indispensable, this approach does not fully account for the marked heterogeneity in disease severity and its progression. In the present study, impaired GLS identified early myocardial dysfunction that was not captured by ejection fraction alone, underscoring the value of myocardial deformation imaging in detecting reduced cardiovascular reserve.

Similarly, reduced coronary flow reserve was observed in nearly one-third of patients and correlated with vegetation size and regurgitation severity. These associations suggest that coronary microvascular dysfunction may reflect a convergence of inflammatory burden, altered blood rheology, and endothelial dysfunction rather than epicardial coronary disease alone. Importantly, CFR provides incremental information beyond structural echocardiographic findings, supporting its role as a sensitive imaging marker of biological vulnerability in IE [9].

From a biological standpoint, coronary flow reserve represents an integrative functional readout of endothelial health, inflammatory microvascular remodeling, altered blood rheology, and impaired metabolic vasodilation. In the setting of infective endocarditis, systemic inflammation, cytokine activation, oxidative stress, and endothelial dysfunction may converge to reduce microvascular adaptability. Accordingly, reduced coronary flow reserve in this cohort should be interpreted as an imaging surrogate of cumulative microvascular and endothelial impairment rather than as a marker of epicardial coronary disease alone.

### 3.3. The Biological Cost of Every Heartbeat

A central concept that emerged from this study is the biological cost of every heartbeat. Although the mean arterial pressure remained relatively stable across age groups, imaging-informed analyses revealed progressive increases in systemic vascular resistance, declining wall shear stress, impaired intravascular transport, and reduced myocardial and microvascular reserve. This dissociation highlights the limitations of conventional hemodynamic measures in capturing the cumulative cardiovascular burden in patients [10].

Each cardiac cycle imposes mechanical, metabolic, and microvascular demands on the myocardium and valvular endothelium [11,12]. Over time, these demands may exceed the adaptive capacity, particularly in the presence of age-related vascular remodeling and microcirculatory impairments [13,14]. In this context, IE may represent not only an infectious process but also the clinical manifestation of a system in which the cumulative biological cost of cardiac work erodes the protective reserves.

### 3.4. Imaging-Derived Cardiovascular Vulnerability

Chronological age alone did not fully capture the heterogeneity of cardiovascular vulnerability in this cohort. In contrast, a constellation of imaging-derived functional, microvascular, and modeling-based hemodynamic indices delineated a cardiovascular vulnerability profile that varied substantially among patients of similar chronological age [15,16]. These findings should not be interpreted as direct measurements of biological age but rather as an integrated imaging phenotype reflecting relative cardiovascular reserve and adaptability under infectious and hemodynamic stress.

Although the present study was not designed to evaluate prognostic prediction, prior studies in cardiovascular and inflammatory diseases have demonstrated that functional and microvascular imaging parameters, including coronary flow reserve and myocardial strain, are associated with adverse clinical outcomes. Within this context, the imaging-derived cardiovascular vulnerability profile described here may represent a biologically plausible substrate underlying disease severity and could warrant prospective evaluation as a prognostic marker in future studies [17,18]. Importantly, such prognostic relevance cannot be inferred from the current data and requires dedicated prospective validation.

### 3.5. Clinical and Imaging Implications

These findings have direct implications for the field of cardiovascular imaging. The incorporation of functional and microvascular imaging markers may enhance early risk stratification in patients with suspected or confirmed infective endocarditis. Patients with preserved blood pressure and modest structural abnormalities may nevertheless have advanced biological vulnerability identifiable by impaired GLS or a reduced CFR [19,20].

From a practical standpoint, advanced echocardiographic assessment may support the early use of transesophageal echocardiography, closer surveillance, and timely multidisciplinary evaluation, including surgical consultation. While these imaging techniques are increasingly available, their integration into routine clinical decision-making will require prospective validation and standardized reproducibility assessment [21].

In clinical practice, imaging-derived functional and microvascular parameters should be viewed as complementary to established structural and clinical criteria rather than as standalone decision tools [22]. For example, reduced coronary flow reserve may help identify patients with limited cardiovascular reserve who could be more vulnerable to hemodynamic stress, potentially informing multidisciplinary discussions regarding monitoring intensity or surgical timing [20]. However, specific thresholds cannot be directly linked to guideline-based indications and require prospective validation before clinical implementation [23,24].

### 3.6. Multimodality Imaging and Metabolic Assessment of Prosthetic Complications

FDG PET/CT was performed in a selected subset of patients with suspected prosthetic valve involvement or inconclusive echocardiographic findings and was evaluated qualitatively. The illustrated PET/CT images (Figure 4 and Figure 5) demonstrate focal or heterogeneous periprosthetic FDG uptake consistent with inflammatory or infectious extension. These findings are presented to illustrate the complementary value of metabolic imaging in complex clinical scenarios rather than to support mechanistic or quantitative biological inference.

### 3.7. Limitations

This study has several limitations. First, its retrospective, single-center design limits causal inference and may introduce selection bias. Coronary angiography and FDG PET/CT were performed in indication-based subgroups and served as complementary mechanistic information rather than systematic assessments.

Second, imaging-derived hemodynamic and vascular indices, including systemic vascular resistance, wall shear stress, and contrast permeability, were calculated using simplified physiological models incorporating assumed cardiac output. These parameters are exploratory and comparative in nature and should not be interpreted as absolute physiological measurements or validated markers of biological aging.

Third, the definition of severe infective endocarditis incorporates both imaging findings and multidisciplinary treatment decisions, introducing incorporation bias. Accordingly, the present analyses describe associations with a clinically adjudicated severity phenotype rather than establishing prognostic prediction or causality.

Fourth, no formal interobserver or intraobserver reproducibility analyses were performed.

Advanced echocardiographic and coronary Doppler measurements are inherently operator- and image-quality dependent, and reported effect sizes and thresholds should be interpreted cautiously.

Fifth, complete-case analysis and the evaluation of multiple imaging parameters without formal correction increase the risk of selection bias and type I error. In addition, unmeasured confounding related to infection severity, antimicrobial regimens, treatment timing, adjunctive cardiovascular therapies, and supportive care may have influenced the observed associations.

Finally, the study lacks direct molecular, biomarker, or histopathological data.

Accordingly, biological interpretations remain inferential and based on established links between infection, inflammation, endothelial dysfunction, and microvascular impairment. Prospective studies with standardized imaging protocols, predefined therapeutic strategies, and longitudinal follow-up are needed to validate these findings and clarify their prognostic relevance.

## 4. Materials and Methods

### 4.1. Study Design and Population

This retrospective observational study included consecutive adult patients diagnosed with definite IE according to the modified Duke criteria and admitted to the “Dr. Carol Davila” Central Military Emergency University Hospital in Bucharest, Romania, between January 2017 and December 2024. Patients with incomplete echocardiographic data or inadequate image quality for advanced functional or coronary Doppler analysis were excluded.

A total of 143 patients met the inclusion criteria and comprised the final study cohort. Clinical, laboratory, echocardiographic, and angiographic data were obtained from institutional medical records. The study was conducted in accordance with the Declaration of Helsinki and approved by the institutional ethics committee. All patients provided written informed consent.

All patients with a diagnosis of definite infective endocarditis according to modified Duke criteria who underwent transthoracic echocardiography during the study period were screened for inclusion. Exclusion criteria included inadequate acoustic windows, hemodynamic instability precluding coronary Doppler acquisition, and incomplete clinical or imaging data relevant to the study objectives.

To enhance consistency, echocardiographic and coronary Doppler examinations were performed according to standardized institutional protocols by experienced operators.

Studies with insufficient image quality for reliable functional or Doppler assessment were excluded at the time of acquisition or during offline analysis.

### 4.2. Echocardiographic Acquisition and Analysis

All patients underwent comprehensive transthoracic echocardiography using high-resolution ultrasound systems, following current American Society of Echocardiography and European Association of Cardiovascular Imaging recommendations. Transesophageal echocardiography was performed when clinically indicated, including suspicion of prosthetic valve involvement, perivalvular complications, or inconclusive transthoracic findings.

Global longitudinal strain was assessed using two-dimensional speckle-tracking echocardiography from standard apical views (four-, two-, and three-chamber) with frame rates optimized according to vendor recommendations. GLS was calculated as the average peak systolic longitudinal strain across all analyzable segments. Studies with inadequate image quality for strain analysis were excluded.

All echocardiographic and Doppler measurements were performed by experienced operators following standardized acquisition protocols. Formal interobserver and intraobserver reproducibility analyses were not performed. This represents an important limitation, particularly for operator- and image-quality-dependent measurements such as coronary flow reserve, global longitudinal strain, and vegetation characterization.

### 4.3. Structural Valve Assessment

Valvular vegetations were characterized by maximal diameter, number, mobility (classified as fixed, moderately mobile, or highly mobile) and echogenicity.

Valvular regurgitation severity was graded using an integrative multiparametric approach.

Left ventricular ejection fraction was measured using the biplane Simpson method.

Global longitudinal strain (GLS) was assessed by two-dimensional speckle-tracking echocardiography from standard apical views and expressed as peak systolic longitudinal strain.

Diastolic function was evaluated using mitral inflow velocities and tissue Doppler imaging to derive the E/e′ ratio, along with indexed left atrial volume. Right ventricular systolic function was assessed using tricuspid annular plane systolic excursion (TAPSE), and pulmonary artery systolic pressure (PASP) was estimated from tricuspid regurgitant velocity.

Coronary microvascular function was assessed noninvasively using transthoracic Doppler echocardiography of the distal left anterior descending coronary artery. Peak diastolic flow velocities were recorded at rest and during pharmacologically induced hyperemia using intravenous adenosine administered according to institutional protocol. Coronary flow reserve was calculated as the ratio of hyperemic to baseline peak diastolic flow velocity. A CFR value < 2.0 was considered indicative of impaired coronary microvascular function. Measurements were performed by experienced operators blinded to clinical outcomes.

### 4.4. Coronary Angiography

Invasive coronary angiography was performed in a predefined, indication-based subgroup of patients for suspected concomitant coronary artery disease, preoperative assessment before valve surgery, unexplained ventricular dysfunction and inconclusive noninvasive coronary evaluation.

Coronary angiography was performed after initial clinical stabilization whenever feasible. Angiographic data were available in 113 patients (79%).

#### Angiographic Analysis and Contrast Transport

Epicardial coronary artery disease was defined as ≥50% luminal diameter stenosis in any major coronary vessel. Beyond anatomical assessment, angiographic sequences were retrospectively analyzed to evaluate contrast transport dynamics using time–density curve analysis.

A contrast permeability index (PCI) was derived by integrating contrast transit time with systemic vascular resistance. This index was used as an imaging-informed surrogate marker of intravascular transport efficiency and endothelial–microvascular interaction. PCI was analyzed as a comparative physiological indicator rather than an absolute quantitative measurement.

### 4.5. Hemodynamic and Metabolic Parameters

Blood pressure was recorded at the time of echocardiographic examination. Mean arterial pressure (MAP) was calculated using standard formulas. Systemic vascular resistance (SVR) was estimated using MAP and assumed cardiac output values adjusted for age and clinical status.

Although valvular pathology may generate complex or turbulent flow patterns, simplified laminar flow assumptions were applied to provide internally consistent, comparative estimates of wall shear stress rather than absolute physiological measurements.

Serum osmolarity was calculated from sodium, glucose, and urea concentrations using established equations. Wall shear stress (WSS) was estimated using simplified laminar flow models incorporating cardiac output and vessel diameter.

These parameters were used to characterize relative biological and hemodynamic burden across age groups and disease severity strata and were interpreted in conjunction with directly measured imaging variables.

Systemic vascular resistance, wall shear stress, and contrast permeability indices were derived using simplified physiological models incorporating measured blood pressure and age-adjusted assumed cardiac output. These parameters were not intended to represent absolute physiological measurements but were used as internally consistent, comparative indices to explore relative hemodynamic and vascular burden across age groups and disease severity strata.

### 4.6. FDG PET/CT Imaging

In selected patients with suspected prosthetic valve involvement or inconclusive echocardiographic findings, FDG PET/CT was performed according to institutional protocols following appropriate patient preparation. PET/CT images were evaluated qualitatively for focal or heterogeneous periprosthetic FDG uptake suggestive of active inflammatory or infectious involvement.

### 4.7. Statistical Analysis

Continuous variables are presented as mean ± standard deviation or median (interquartile range), as appropriate. Correlations between imaging, hemodynamic, and microvascular parameters were assessed using Pearson or Spearman correlation coefficients, as appropriate.

Multivariable linear and logistic regression analyses were performed to identify imaging parameters independently associated with disease severity and perivalvular complications. Variables were selected based on clinical relevance and univariable association. Model discrimination was assessed using receiver operating characteristic curve analysis.

Baseline comorbidities, including diabetes mellitus, hypertension, coronary artery disease, and chronic kidney disease, were systematically recorded and considered in multivariable analyses when clinically relevant and available.

A two-sided *p* value < 0.05 was considered statistically significant. Statistical analyses were performed using SPSS version 26 (IBM Corp., Armonk, NY, USA).

Variables entered into multivariable regression models were selected a priori based on clinical relevance and univariable association with outcomes (*p* < 0.10). Collinearity was assessed before model construction. Results are reported as odds ratios with 95% confidence intervals. The association between coronary flow reserve and disease severity remained significant after adjustment for vegetation size.

Analyses were performed using complete-case methodology. No imputation for missing data was performed.

Given the retrospective design and heterogeneous availability of advanced imaging parameters, analyses were performed using a complete-case approach. This strategy was chosen to preserve internal consistency of multivariable models but may introduce bias if missingness is related to disease severity, hemodynamic instability, or image quality. No formal correction for multiple comparisons was applied due to the exploratory nature of the analyses. Accordingly, reported associations should be interpreted as hypothesis-generating rather than confirmatory. Multivariable models were constructed using variables selected a priori based on clinical relevance and univariable association (*p* < 0.10). Collinearity was assessed before model entry.

### 4.8. Outcome Definitions

Severe infective endocarditis was defined as the presence of at least one of the following during index hospitalization:

1. perivalvular complications (abscess, aneurysms, prosthetic dehiscence, or leaflet perforation) detected by transesophageal echocardiography or cardiac computed tomography;
2. severe valvular regurgitation requiring surgical intervention; or
3. clinical indication for urgent or emergent valve surgery as determined by the multidisciplinary endocarditis team.

Perivalvular complications were defined according to current guideline criteria and confirmed by transesophageal echocardiography, with cardiac computed tomography used when clinically indicated.

### 4.9. Ethics

The study protocol was approved by the Ethics Committee of the “Dr. Carol Davila” Central Military Emergency University Hospital (Decision No. 841/17 December 2025). Written informed consent was obtained from all patients included in the study prior to data collection and analysis.

## 5. Conclusions

The severity of infective endocarditis reflects more than just structural valve pathology or microbial exposure. Advanced echocardiographic and coronary Doppler imaging reveal a spectrum of biological cardiovascular vulnerabilities characterized by impaired myocardial deformation, microvascular dysfunction, and reduced transport efficiency despite preserved conventional hemodynamic measures. These findings support the concept that the cumulative biological cost of each heartbeat shapes the disease expression and progression in infective endocarditis. The integration of functional and microvascular imaging into routine assessment may improve early detection, refine risk stratification, and guide timely intervention.

These findings should be interpreted as exploratory and hypothesis-generating. While the observed associations are internally consistent and biologically plausible, they require prospective validation in adequately powered studies with predefined statistical frameworks.

### Perspectives

**Competency in Medical Knowledge**

Infective endocarditis severity is not fully explained by structural valve abnormalities alone. Advanced echocardiographic and coronary Doppler imaging can identify subclinical myocardial, hemodynamic, and microvascular vulnerability that is associated with disease severity and perivalvular complications despite preserved conventional hemodynamic parameters.

**Translational Outlook**

Prospective studies are needed to determine whether integration of functional and microvascular imaging markers into routine evaluation of suspected infective endocarditis can improve early risk stratification, optimize timing of transesophageal echocardiography, and guide multidisciplinary management strategies.

## Figures and Tables

**Table 1:** Baseline Characteristics of the Study Population (n = 143).

Parameter	Median (IQR)	Range	%/n
Age (years)	67 (59–74)	23–95	–
Sex (M/F)	–	–	73% (104)/27% (39)
Heart rate (bpm)	75 ± 18	31–145	–

**Table 2:** Baseline comorbidities according to infective endocarditis severity.

Comorbidity	Overall Cohort (n = 143)	Severe IE Phenotype (n = 112)	Non-Severe IE Phenotype (n = 31)	p * Value
Diabetes mellitus	42 (29%)	18 (16.1%)	24 (77.4%)	<0.01
Hypertension	78 (55%)	34 (35.7%)	11 (35.5%)	<0.01
Coronary artery disease	31 (22%)	17 (15.2%)	14 (45.2%)	<0.03
Chronic kidney disease (eGFR < 60 mL/min/1.73 m^2^)	36 (25%)	21 (18.8%)	15 (48.4%)	<0.01
Atrial fibrillation	27 (19%)	16 (14.3%)	11 (35.5%)	<0.02
Prior heart failure	33 (23%)	20 (17.9%)	13 (41.9%)	<0.01
Prior valvular heart disease	46 (32%)	28 (25.0%)	18 (58.1%)	<0.02
Peripheral arterial disease	14 (10%)	9 (8.0%)	5 (16.1%)	<0.03
Chronic obstructive pulmonary disease	18 (13%)	10 (8.9%)	8 (25.8%)	<0.03
Active malignancy	9 (6%)	5 (4.5%)	4 (12.9%)	<0.03

* *p* values were derived using χ^2^ or Fisher’s exact tests, as appropriate, based on cell counts.

**Table 3:** Baseline Demographic, Clinical, and Laboratory Characteristics of the Study Population.

Variable	Overall Cohort (n = 143)
Demographics	
Age, years, median (IQR)	67 (59–74)
Body mass index (BMI), kg/m^2^, median (IQR)	26.1 (23.4–29.8)
Risk Factors for Infective Endocarditis	
Intravenous drug use, n (%)	29 (20%)
Tattoos, n (%)	34 (24%)
Prosthetic valve, n (%)	51 (36%)
Infectious/Immunological Status	
HIV infection, n (%)	7 (5%)
Chronic immunosuppression, n (%)	16 (11%)
Active malignancy, n (%)	9 (6%)
Cardiovascular Comorbidities	
Hypertension, n (%)	78 (55%)
Diabetes mellitus, n (%)	42 (29%)
Coronary artery disease, n (%)	31 (22%)
Atrial fibrillation, n (%)	27 (19%)
Non-Cardiovascular Comorbidities	
Chronic kidney disease (eGFR < 60 mL/min/1.73 m^2^), n (%)	36 (25%)
Chronic obstructive pulmonary disease, n (%)	18 (13%)
Chronic liver disease, n (%)	11 (8%)
Baseline Laboratory Parameters	
Hemoglobin, g/dL, mean ± SD	10.8 ± 2.1
White blood cell count, ×10^9^/L, median (IQR)	12.4 (9.6–16.8)
C-reactive protein, mg/L, median (IQR)	78 (34–162)
Serum creatinine, mg/dL, median (IQR)	1.3 (0.9–1.9)
Estimated GFR, mL/min/1.73 m^2^, median (IQR)	62 (45–82)
Metabolic Panel	
Fasting glucose, mg/dL, median (IQR)	118 (98–156)
Total cholesterol, mg/dL, mean ± SD	172 ± 38
LDL cholesterol, mg/dL, mean ± SD	102 ± 31
HDL cholesterol, mg/dL, mean ± SD	44 ± 12
Triglycerides, mg/dL, median (IQR)	138 (96–192)
Endocrine Parameters	
Thyroid-stimulating hormone (TSH), mIU/L, median (IQR)	1.9 (1.2–2.8)

**Table 4:** Structural Echocardiographic Characteristics of Valvular Vegetations.

Parameter	Mean ± SD	Range	%/n
Vegetation size (cm)	1.58 ± 0.67	0.31–2.47	–
Number of vegetations	–	–	1: 61%/2: 29%/≥3: 10%
Vegetation mobility	–	–	High: 52%/Moderate: 35%/Fixed: 13%
Vegetation echogenicity	–	–	Hyperechogenic: 45%/Mixed: 38%/Hypoechogenic: 17%

**Table 5:** Functional Echocardiographic and Hemodynamic Parameters.

Parameter	Mean ± SD	Range
LVEF (%)	48 ± 13	30–70
GLS (%)	−16.9 ± 3.5	−10 to −24
E/e′ ratio	15.8 ± 7.1	5.3–39
Indexed LAV (mL/m^2^)	42 ± 15	18–78
TAPSE (mm)	18.7 ± 4.7	12.4–28
PASP (mmHg)	44 ± 17	18–78

**Table 6:** Coronary Microvascular Parameters.

Parameter	Mean ± SD	Range
LAD flow (mL/s)	44 ± 18	15–329
CFR	2.2 ± 0.8	0.9–4.1

**Table 7:** Perivalvular Complications and Associated Vegetation Size.

Complication	% of Patients	Vegetation Size (cm)
Abscess	38%	2.1 ± 0.4
Aneurysm	25%	2.0 ± 0.5
Prosthetic dehiscence	18%	2.0 ± 0.6
Leaflet perforation	14%	1.9 ± 0.5

**Table 8:** Correlations Between Key Imaging Parameters.

Parameter 1	Parameter 2	r	*p* Value
Vegetation size	Regurgitation grade	0.62	<0.001
Vegetation size	PASP	0.41	0.003
GLS	E/e′	−0.58	<0.001
CFR	Vegetation size	−0.39	0.002
CFR	Regurgitation grade	−0.36	0.004

**Table 9:** Blood Pressure and Mean Arterial Pressure by Age Group.

Age Group (Years)	n	Mean SBP (mmHg)	Mean DBP (mmHg)	MAP (mmHg)
23–30	5	127.2	72.8	90.9
31–40	7	135.1	72.7	93.5
41–50	9	120.7	80.2	93.7
51–60	20	126.9	72.0	90.3
61–70	43	127.9	73.5	91.6
71–80	48	127.9	73.1	91.3
>80	6	122.0	72.3	88.9

**Table 10:** Systemic Vascular Resistance by Age Group.

Age Group (Years)	n	MAP (mmHg)	Assumed CO (L/min)	SVR (dyn·s·cm^−5^)
23–30	5	90.9	5.6	1227.1
31–40	7	93.5	5.4	1311.1
41–50	9	93.7	5.2	1364.6
51–60	20	90.3	5.0	1364.8
61–70	43	91.6	4.8	1443.3
71–80	48	91.3	4.6	1500.9
>80	6	88.9	4.3	1560.9

**Table 11:** Calculated Serum Osmolarity by Age Group.

Age Group (Years)	n	Serum Osmolarity (mOsm/kg)
23–30	5	289.3
31–40	7	292.2
41–50	9	295.8
51–60	20	297.3
61–70	43	307.8
71–80	48	292.8
>80	6	293.2

**Table 12:** Estimated Wall Shear Stress by Age Group.

Age Group (Years)	n	Estimated WSS (dyn/cm^2^)
23–30	5	≈17.0
31–40	7	≈14.7
41–50	9	≈12.8
51–60	20	≈11.0
61–70	43	≈9.5
71–80	48	≈8.3
>80	6	≈6.9

**Table 13:** Contrast Transport Dynamics and Permeability Index.

Age Group (Years)	n	Velocity (cm/s)	Transit Time (s)	PCI (×10^−4^)
23–30	5	38	0.47	7.1
31–40	7	36	0.50	6.1
41–50	9	34	0.53	5.4
51–60	20	32	0.56	5.0
61–70	43	30	0.60	4.3
71–80	48	28	0.64	3.7
>80	6	26	0.69	3.2

**Table 14:** Age-Stratified Right Ventricular and Coronary Doppler Parameters.

Age Group (Years)	n	PASP (mmHg)	TAPSE (mm)	LAD Flow (mL/s)	CFR
23–30	5	32	21.5	52.0	2.80
31–40	7	36	20.5	49.0	2.60
41–50	9	40	19.5	47.0	2.40
51–60	20	43	18.8	45.0	2.25
61–70	43	45	18.4	43.5	2.15
71–80	48	47	18.0	41.5	2.05
>80	6	50	17.0	39.5	1.90

## Data Availability

The data presented in this study are available upon request. The data are not publicly available because of the confidentiality of health data.

## Abbreviations

IE, infective endocarditis; CFR, coronary flow reserve; GLS, global longitudinal strain; SVR, systemic vascular resistance; WSS, wall shear stress; PET/CT, positron emission tomography/computed tomography; FDG, fluorodeoxyglucose; TTE, transthoracic echocardiography; TEE, transesophageal echocardiography; MAP, mean arterial pressure; LV, left ventricle; LVEF, left ventricular ejection fraction; CKD, chronic kidney disease; COPD, chronic obstructive pulmonary disease; IQR, interquartile range.
